# Pharmacogenomic testing implementation: Tertiary care center experience and results of a pilot of 512 patients

**DOI:** 10.1016/j.gimo.2025.103426

**Published:** 2025-03-22

**Authors:** Dana Bakheet, Hana Al Alshaykh, Ghadi Askar, Sateesh Maddirevula, Samya Hagos, Nasir Hamad Alshahrani, Midrar Alhusseini, Amani Moharram, Faries Algadhi, Osama Alswailim, Moatasem Alsulaim, Bandar Alamro, Fadl Elmula Mohamed Fadl Elmula, Bahadur Khan, Ahmed Almustafa, Ahmed Alshehri, Dimpna Brotons, Fahad Alajlan, Mohammed Alnahedh, Abdullah Alsuwaidan, Salah Baz, Ahmed Alfares

**Affiliations:** 1 King Faisal Specialist Hospital and Research Centre, Riyadh, Saudi Arabia; 2 College of Medicine, Alfaisal University, Riyadh, Saudi Arabia

**Keywords:** Genomics, Personalized medicine, Pharmacogenetics (PGx), Pharmacogenomics (PGx), Precision medicine

## Abstract

**Purpose:**

Pharmacogenomic (PGx) testing has proven significant clinical utility in minimizing adverse drug reactions and maximizing therapeutic effects. This report is a proof of concept of the clinical implementation of PGx testing at a tertiary care hospital in Riyadh, Saudi Arabia.

**Methods:**

In collaboration with different departments, we implemented the PGx testing clinical service into our electronic health record cerner for heart and neurology centers patients. We used the PharmacoScan microarray-based test to perform genotyping.

**Results:**

To date, 512 patients have been tested. 97.5% of all patients have at least 1 gene with altered function. Among those patients, *n* = 402 (78.5%) had 3 or more genes with altered function (3 genes = 197, 38.5%, 4 genes = 134, 26.2%, 5 genes = 58, 11.3%, and 6 genes = 13, 2.5%).

**Conclusion:**

Our work describes the successful implementation of PGx testing in clinical practice and encourages further research on improving patient outcomes. Moreover, we describe the major challenges at each step of our approach, which provides our institute and others with lessons and insights on implementing PGx testing into clinical practice.

## Introduction

Pharmacogenomics (PGx) aims to define the genetic variants that influence drug efficacy and/or toxicity.^1^^,^^2^ Single-nucleotide variations (SNPs) in germline regions that affect drug response are common in the human population. A typical scenario affecting drug pharmacokinetics occurs when administering prodrugs, SNPs that result in loss-of-function variants affecting the metabolizing enzyme can decrease or block drug action. An example of this scenario is the drug clopidogrel and its conversion to the active form 2-oxo-clopidogrel.^2^ In contrast, multiple adverse drug reactions (ADRs) are a result of SNPs affecting drug pharmacodynamics; these include malignant hyperthermia due to exposure to inhaled anesthetics and others.^3^ Multiple large-cohort studies estimated that the carriage status type of at least 1 actionable pharmacogenes is 91% to 99.75%.^4^, ^5^, ^6^ A study found that approximately 50% of prescriptions in the United States are affected by actionable germline pharmacogenes,^7^ whereas local data in Saudi Arabia were not significantly different, 46%.^8^ Efficacy rates of drugs have been reported to vary between 25 and 80% among individuals,^9^ and it was found that for every 1 person helped, there are 3 to 24 individuals who have failed to show response.^10^ In terms of safety, ADRs can affect up to 15% of hospitalized patients.^11^ In Saudi Arabia, a study conducted in 2022 evaluating 2349 ADR reports found that most reported drugs were antimicrobial drugs (26.9%), hematologic drugs (19.7%), and neuropsychiatric drugs (12.9%), a big proportion of those drugs having PGx implications.^12^ Utilizing PGx testing can lead to better patient outcomes and better utilization of health care system resources over the long run.^11^^,^^13^, ^14^, ^15^

PGxs implementation into clinical practice is a complex process that requires multidisciplinary collaboration and stakeholder engagement. Many institutes worldwide have adopted PGxs testing in clinical practice and reported their experiences and challenges to implementation.^4^^,^^16^, ^17^, ^18^, ^19^, ^20^, ^21^, ^22^, ^23^, ^24^

Although numerous studies in the Middle East have been conducted to characterize the unique genetic makeup of this diverse population and specifically the actionable pharmacogenomic variation relating to different gene-drug pairs, no studies have reported the implementation of PGx testing into clinical practice.^25^, ^26^, ^27^, ^28^, ^29^, ^30^, ^31^, ^32^, ^33^, ^34^, ^35^

Multiple international studies describe the steps and stakeholders involved in building a PGxs service at your institution.^16^^,^^36^ According to Kabbani et al,^16^ implementing a PGx testing service in clinical practice requires at least 8 main stakeholders. The factors include drug regulators authorizing or requiring specific PGx tests, hospital leadership supporting the process, a pharmacy and therapeutics committee leading the process in collaboration with the hospital, molecular laboratory and information technology, and health care providers (HCPs) ordering the test, understanding the results, making the appropriate therapeutic decisions, and explaining them to the patients.^16^ The implementation process starts with forming the implementing team, mainly consisting of the stakeholders mentioned above, identifying the target setting and patients, selecting gene-drug pairs satisfying the accepted evidence threshold, building service infrastructure, delivering education for providers and patients, and providing clinical and technical support all along.^16^^,^^36^

This research article outlines the multilevel implementation process of PGx testing at a tertiary care hospital, highlighting the facilitators and obstacles encountered. Additionally, it presents proof-of-concept results from a cohort of 512 cardiology and neurology patients.

## Materials and Methods

### Setting and inclusion criteria

This report describes the pilot phase of clinical implementation of PGx testing at a tertiary care hospital in Riyadh, Saudi Arabia, and the results of the patients tested in this pilot phase. The test was implemented within the electronic health record (EHR) as a regular laboratory test.

During the initial planning phase, we conducted a comprehensive literature review to gain insights from other centers that have successfully implemented PGx testing. Our approach involved multiple concurrent pathways, addressing management, technical considerations, stakeholder engagement, and education and awareness.

In the management aspect, we secured project approvals from senior leadership. We also engaged various stakeholders within our institution, ensuring that they were integrated into the process. Simultaneously, we evaluated evidence, assessed our technical and informatics capabilities, and organized educational sessions for HCPs.

Five prescribing HCPs were included from the heart center and 5 from the stroke team—neurology department—for this pilot phase, and they were the champions leading the implementation. All prescribing HCPs were cardiology and neurology consultants. The PGx testing was launched in September 2023 and was offered for cardiology or neurology adult and pediatric patients seen at our institute and meeting the below eligibility criteria:

1. On or will be on a medication affected by PGx: metoprolol, simvastatin, atorvastatin, clopidogrel, flecainide, or warfarin.
2. In response to suspected drug-induced adverse effects, simvastatin and atorvastatin.
3. In response to therapy failure, clopidogrel has been defined as the occurrence of a thrombotic event/ischemic event during clopidogrel therapy in patients with heightened platelet reactivity.^37^

Institutional Review Board approval was obtained for conducting this study. Patient written consent was not collected when performing this test because it was a clinical test part of their management approach. However, verbal consent was collected from every patient per our hospital practice and policy for genetic tests that are part of care.

### Selected drugs

The implementation of the PGx testing pilot was initially started in collaboration with the heart center and then expanded to include the ischemic stroke team from the neurology department because of the overlap between the inclusion criteria that allows for accepting more patients. Patients from inpatient and outpatient settings meeting the inclusion criteria were eligible for PGx testing. The medications included are formulary medications used for cardiac or cerebrovascular indications and supported by high-level evidence defined as Clinical Pharmacogenetics Implementation Consortium (CPIC) level-A and/or PharmGKB 1A. Table 1 shows the included medications, their corresponding genes, and actionable phenotypes.

**Table 1:** Selected medications, their corresponding genes, and actionable phenotypes

Medication (Therapeutic Category)	Gene	Actionable Phenotype	Clinical Action
Metoprolol (beta blocker)	*CYP2D6*	Ultrarapid metabolizer Intermediate metabolizer Poor metabolizer	Adjust dose or consider adjusting dose
Simvastatin (lipid-lowering drug)	*SLCO1B1*	Decreased function Poor function	Adjust dose or use an alternative agent
Atorvastatin (lipid-lowering drug)	*SLCO1B1*	Decreased function Poor function	Adjust dose or use an alternative agent
Flecainide (antiarrhythmic)	*CYP2D6*	Ultrarapid metabolized Intermediate metabolizer Poor metabolizer	Adjust dose
Clopidogrel (anti-platelet)	*CYP2C19*	Intermediate metabolizer Poor metabolizer	Avoid
Warfarin (anticoagulant)	*VKORC1*, *CYP4F2*, and *CYP2C9*		Refer to warfarin calculator to calculate initial dose

Actionable phenotype: phenotype that is expected to alter medication response affected by the that gene and is associated with a therapeutic recommendation to modify the medication regimen. Flecainide: we have utilized Dutch Pharmacogenetics Working Group recommendations for this medication.

### Genotyping approach

PGx testing was performed at the College of American Physicians (CAP) Accredited Center for Genomic Medicine (CGM) at our institute. The PharmacoScan microarray-based assay was used to genotype the extracted DNA from venous blood samples. DNA is extracted as described in Monies et al.^38^ The PharmacoScan assay includes genome-wide and pharmacogene coverage and is a clinically validated and commercially available test.^39^ When compared with other array-based tests, PharmacoScan had the highest pick-up of PGx actionable genes (124 genes) and copy-number variation calling.^40^ The resultant data are then analyzed using AxiomTM Analysis Suite (v5.2.0.65), and diplotype to phenotype translation is performed using the metabolizer library file.^41^ In certain instances, the software produced multiple potential phenotypes or a range of phenotypes for a single result. In these cases, we revisited the genotype and interpreted it according to CPIC guidelines. We ran CAP proficiency samples to validate the pipeline, achieving 100% concordance with CAP survey results before launching our test.

### Data curation

Phenotype calls were approved because they were reported from the AxiomTM Analysis Suite with diplotype to phenotype translation.^41^ PGxs-guided prescribing recommendations were adopted directly from CPIC. Before upload into EHR, results were reviewed by a pharmacist and verified by a geneticist.

### Integration into EHRs

The Healthcare Information Technology Affairs (HITA) team developed an order within the EHR called “CGM Pharmacogenomic Profiling,” allowing physicians to order PGxs testing. Currently, results are entered manually into the EHR as discrete variables. A clinical scientist enters the results, which are then reviewed and verified by a consultant geneticist. Once the results are verified, physicians are notified via email that the test results for their patients are available.

In the flowsheet that patient test results are usually posted, a new section was built and named “Pharmacogenomic profiling,” which consists of 2 fields for each gene: gene name and the phenotype. For *CYP2C9* and *CYP4F2*, results are displayed as diplotypes with star allele nomenclature (eg, ∗1/∗1, ∗1/∗9, etc), *VKORC1* is provided as either A/A, A/G, or G/G to aid for the utilization of test results in warfarin dosing online calculator (http://www.warfarindosing.org/). In contrast, the results for *SLCO1B1*, *CYP2D6*, and *CYP2C19* are displayed as metabolizer status.

In the comments section of each gene, a list of all possible interpretations for each phenotype is provided based on gene-drug pairs. To improve utilization of PGxs testing, we developed pre- and posttest passive and active clinical decision support (CDS) alerts for clopidogrel, metoprolol, flecainide, warfarin, simvastatin, and atorvastatin. The pretest CDS (passive alerts) alerts fire when any of the included medications in this pilot is ordered, given that the patients do not have a previous PGx test result. Posttest CDS alerts fire when placing a new medication order while the patient has an actionable PGx test result (as per the definition under Table 2) that warrants regimen adjustment (active alerts). These alerts allow physicians to order the PGx test, modify the medication order, or override the alert if clinically indicated.

**Table 2:** Summary data for *n* = 512 patients and 6 tested genes

Gene-Drug Pair	Normal Results (Frequency, Percent)	Altered-Function Results (Frequency, Percent)	Alterations Requiring Clinical Action (Frequency, Percent)	Most Common Diplotypes (Diplotype, Percent)
*CYP4F2*-Warfarin	124, 24.2	388, 75.7	NA	∗1/∗3, 28.5
*CYP2C19*-Clopidogrel	190, 37.1	322, 62.8	123, 24	∗1/∗1, 36.7
*SLCO1B1*-Statins	188, 36.7	324, 63.2	212, 41.4	∗1A/∗1A, 24.4
*VKORC1*-Warfarin	269, 52.5	243, 47.4	NA	A/G, 43.2
*CYP2D6*-Metoprolol	298, 58.2	214, 41.7	174, 33.9	1/∗1, 13.1
*CYP2C9*-Warfarin	327, 63.8	185, 36.1	NA	∗1/∗1, 62.3

NA: *CYP2C9*, *VKORC1*, and *CYP4F2* data were not included in the column of “Alterations requiring clinical action” because acquiring this knowledge requires a calculation of the results of the 3 genes together.

*NA*, not applicable.

Medication-specific recommendations and dosing instructions can be acquired through the implemented CDS alerts within the EHR, in which posttest PGx alerts will appear containing specific recommendations when the patient has actionable results. Additionally, further instructions can be acquired by consulting with a PGxs pharmacy specialist or by referring to the providers’ tip sheet provided to the prescribers.

After PGx test results are returned and posted in the EHR, providers review the results and are responsible for applying modifications to medications affected by the genes included on the test menu if the results are actionable. As of the time of writing this report, test results were shared with patients through our patient portal, the Altakhassusi App, from which patients could access them within the lab results section. Meanwhile, the PGxs clinic was being developed to be operated by a PGxs pharmacy specialist, offering a setting for patients to learn about PGxs, understand the results of their PGx testing, and comprehend its implications on their current, past, and possibly future medications.

### HCP education

HCP education was provided by a PGxs pharmacy specialist, who is a professional holding a Doctor of Pharmacy degree, has completed a postgraduate year 2 pharmacy specialty residency in PGxs accredited by the American Society of Health-System Pharmacists, and holds a valid Pharmacotherapy Specialty Certification. Participating providers received a structured 1-hour in-person session during which the PGxs pharmacy specialist delivered information on PGxs, the role of testing in improved patient outcomes, and then guided them through a tutorial on how to order the test, navigate the test results in EHR, interpret results, and apply PGxs-guided medication adjustment as clinically indicated. Providers were also provided with a PDF tip sheet on how to interpret the test results and have the prescribing information handy when needed. The PGxs pharmacy specialist participated in department grand rounds and followed up regularly with providers through short in-person meetings and emails to address questions and gather feedback to improve the process. Additionally, the HITA team provided a live tutorial to participating providers on how to order the test and navigate test results in the EHR and shared a PDF tutorial with providers.

Throughout the implementation phase, multiple meetings were held to obtain providers’ feedback. Overall, their feedback was positive in terms of the value of PGx testing in personalizing medication prescribing and clarity in the language used for interpreting PGx test results. Some constructive feedback was obtained to improve the testing process and results notifications.

## Results

Up to date, PGx profiling has been done for *n* = 512 patients, with data showing results for 6 genes for each patient: *CYP2C19*, *CYP2C9*, *CYP2D6*, *CYP4F2*, *SLCO1B1*, and *VKORC1*. Our patient cohort comprised 325 males (63.5%) and 187 females (36.5%). The age range of patients spanned from 2 to 95 years, with fewer than 4% being pediatric patients (*n* = 16). The largest proportion of patients fell within the 61 to 80 year age group (*n* = 224, 43.7%), followed by the 41 to 60 year range (*n* = 190, 37.1%). The remaining patients were distributed across the 19 to 40 year age group (*n* = 68, 13.2%), those under 18 years (*n* = 16, 3.1%), and the 81 to 100 year range (*n* = 14, 2.7%). We categorized the phenotypes/genotypes into normal results (genotypes conferring a normal metabolizer status) and results with altered gene function (genotypes conferring all other metabolizer statuses including ultrarapid, rapid, intermediate, poor, and indeterminate metabolizers). Table 2 summarizes our patient cohort and the 6 tested genes.

**Figure 1:**
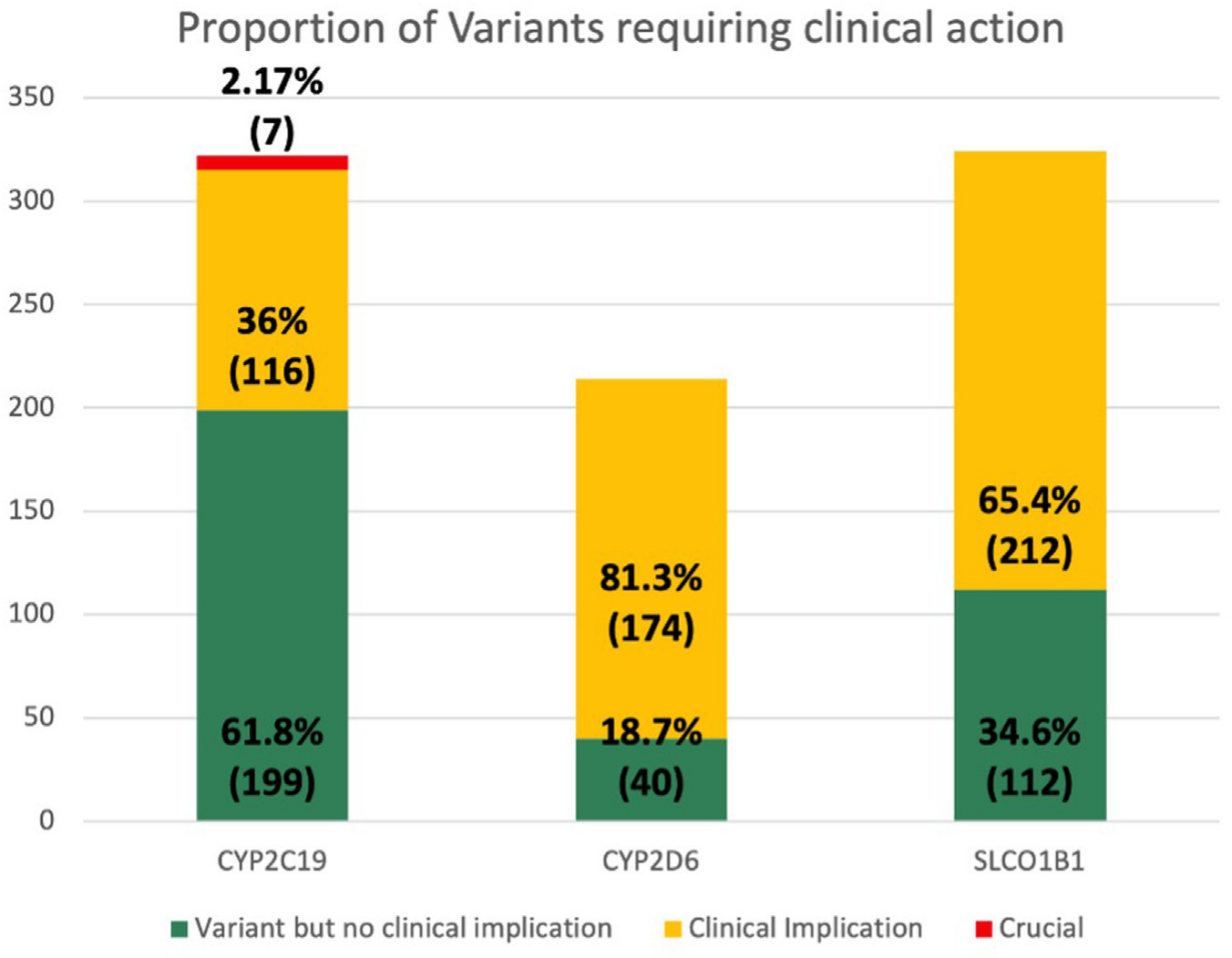
**Proportion of patients with altered function results with clinical recommendations**.

We observed the highest number of normal results in the gene *CYP2C9* (*n* = 327, 63.8%) and the lowest in the gene *CYP4F2* (*n* = 124, 24.2%). In terms of clinical recommendations, not all patients have clinical recommendations, either because their phenotype/genotype is treated with standard medications/dosing or because no guidelines are available for them at this point. The *SLCO1B1* gene had the highest number of patients requiring clinical action in relation to statins (*n* = 212, 41.4%). The proportion of patients requiring clinical action is displayed in Figure 1.

**Figure 2:**
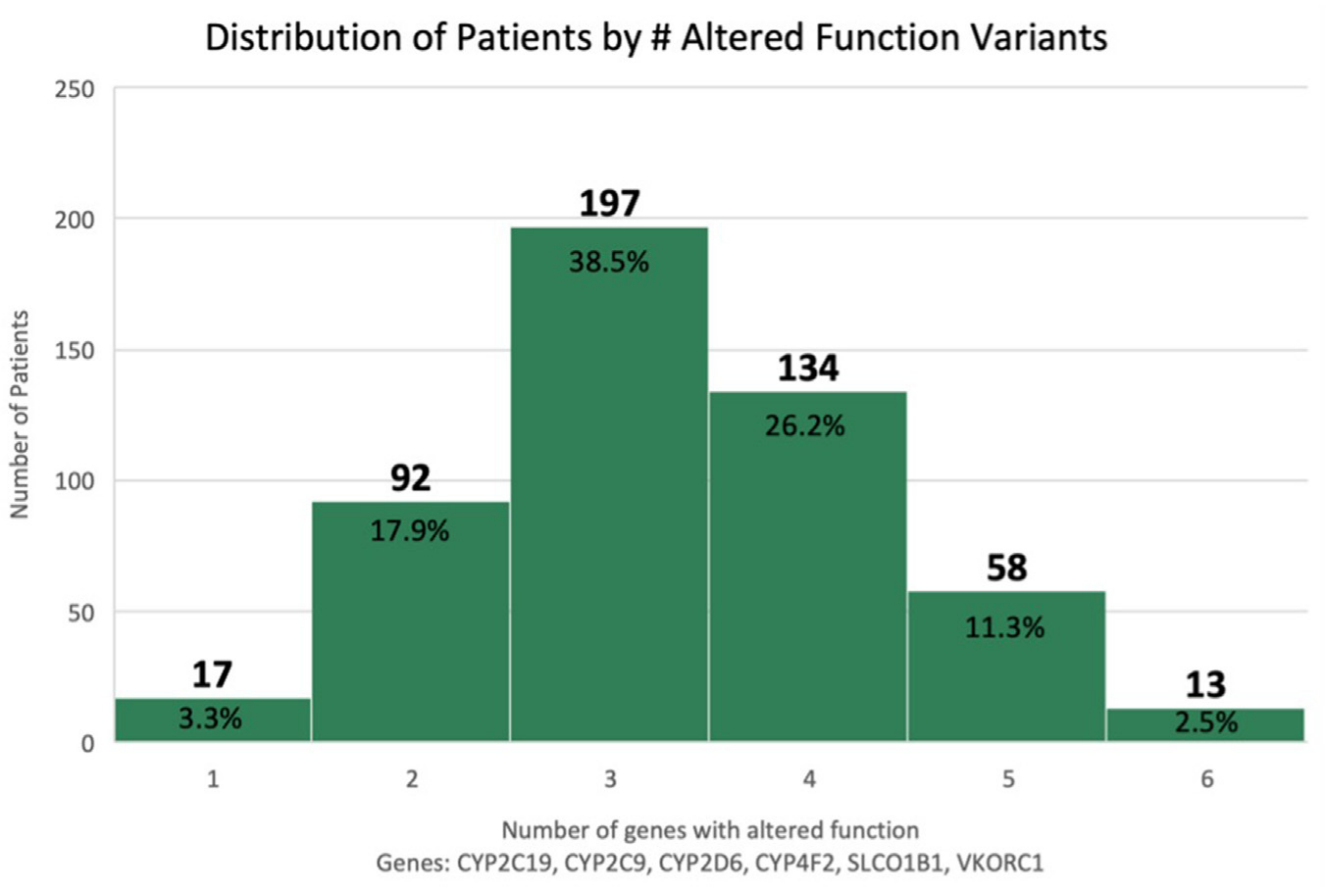
**Distribution of patients by the number of altered function variants**.

Among the 512 patients, 402 (78.5%) had 3 or more genes with altered function (3 genes = 197, 38.5%, 4 genes = 134, 26.2%, 5 genes = 58, 11.3%, and 6 genes = 13, 2.5%). Figure 2 shows the distribution of patients by the number of altered-function genes.

## Discussion

At the start of this initiative, we assembled an interdisciplinary team that built the in-house infrastructure and service. The collaborative efforts of healthcare professionals from diverse fields, involved at every stage, were critical to the program’s successful adoption. Our core team comprised scientists from the Centre for Genomic Medicine, a PGx specialist, consultants in cardiology and neurology, and the HITA team. The Centre for Genomic Medicine was the initiative owner in this dynamic. They were responsible for planning the logistics and technical aspects of the implementation process, including which sequencing technology and commercial kits to use. The PGx specialist and the CGM members handled the literature review, decisions on gene-drug pairs, patient result interpretation, and ongoing updates of the guidelines integrated into the EHR.

Additionally, the PGx specialist conducted regular educational sessions for HCPs. The HITA team played a crucial role in developing the EHR infrastructure, including creating test order commands, displaying results for specific gene-drug pairs, aligning results with clinical recommendations, and building pre- and posttest CDS alerts. The proactive engagement of physicians during the implementation and deployment phases contributed to having a robust environment, in which HCPs were involved in the decision-making process by providing their dynamic feedback on included gene-drug pairs, results layout and accessibility, guidelines accessibility, and refining the design and content of our CDS alerts. This dedicated team was instrumental in promptly gaining knowledge, efficiently troubleshooting problems, and integrating the necessary skills and expertise for a successful implementation.

Despite our progress, we encountered several limitations, highlighting key lessons for the next PGx testing and reporting expansion phase. One significant challenge involved our genotyping kit, which occasionally returned multiple potential outcomes because of phase ambiguity. In such instances, patients’ phenotype/genotype results were reported as indeterminate. We are upgrading our genotyping kit to address this issue in preparation for the next phase.

From an information technology perspective, the manual entry of test results was a significant area of improvement to accommodate the large volume of PGx testing because the service is anticipated to grow across the hospital. A major improvement for enhancing PGx testing implementation is automated result reporting into the EHR and the design of CDS tools to aid clinical practice. Various models have been described in the literature.^42^^,^^43^ We have implemented pretest and posttest CDS alerts to encourage providers to order PGx tests when necessary and to provide recommendations based on genetic findings. However, we faced challenges with HCPs’ responsiveness to these alerts and their follow-up actions after the release of test results. This aligns with challenges reported in numerous studies.^44^, ^45^, ^46^ Additionally, our system lacks integrated notification features, requiring us to rely on email alerts to notify HCPs when results are available.

Furthermore, there is a recognized need for more frequent and structured educational sessions to enhance provider engagement and understanding of PGx testing.

Although our pilot study was small, our findings aligned with variant distributions observed in larger regional cohorts. For example, a study of a Saudi population (*n* = 1928) reported a high prevalence of the 1 of 15 variant in *SLOC1B1* (21.32%). Similarly, the *CYP2C9* ∗2 variant was common (20.1%), influencing warfarin dosing.^47^ Studies in other populations in the region also reported identical distribution. A large data-mining study on 136,129 patients concluded that *CYP2D6* affects 7 of 33 top-dispensed drugs. This study concluded that 38.5% of patients have altered function associated with *CYP2D6*.^27^

As PGxs evolves, new star alleles are discovered, and the function of existing alleles may be redefined (eg, an uncertain function star allele may be changed to decreased function, based on new evidence). Although we currently utilize genetic studies from Western countries, there is a clear need for multicenter research in the Middle East to ensure applicability to our population. As one of the few CAP-accredited centers offering PGx testing in the region, we are strategically positioned to establish a biobank that integrates clinical and genetic data. This resource aims to characterize the unique genetic landscape of our underrepresented population, thereby addressing knowledge gaps and enhancing our understanding of genetic influences on pharmacotherapy responses.

To our knowledge, aside from a few research projects and a clinical feasibility study, our center is the first in the Middle East North Africa region to successfully launch PGx testing as a clinical service^25^, ^26^, ^27^, ^28^, ^29^, ^30^, ^31^, ^32^, ^33^, ^34^, ^35^

### Future directions

The implementation of PGx in any institution represents a substantial undertaking demanding continuous development and updates. Our institution aims to extend the service to all clinical departments, prioritizing clinical services with higher urgency for PGxs implementation, including transplant and oncology. As for reported genes, we will add them based on clinical needs within each clinical service and the availability of level-A guidelines. Additionally, we anticipate completing the automation of result reporting within 1 year, enabling us to report on a broader range of gene-drug pairs and establish a comprehensive biobank integrating patients’ genetic and clinical data. As the project advances, we plan to progressively transition toward preemptive PGx testing for all patients in our institute, ultimately aspiring to serve as a national and regional center for preemptive PGx testing.

## Data Availability

The data supporting this study’s findings are available from by request from the corresponding author, under the support of 10.13039/501100002382King Faisal Specialist Hospital and Research center.

## Conflict of Interest

The authors declare no conflicts of interest.
